# Aqueous Extract of *Gracilaria tenuistipitata* Suppresses LPS-Induced NF-κB and MAPK Activation in RAW 264.7 and Rat Peritoneal Macrophages and Exerts Hepatoprotective Effects on Carbon Tetrachloride-Treated Rat

**DOI:** 10.1371/journal.pone.0086557

**Published:** 2014-01-27

**Authors:** Chin-Kai Tseng, Chun-Kuang Lin, Hsueh-Wei Chang, Yu-Hsuan Wu, Feng-Lin Yen, Fang-Rong Chang, Wei-Chun Chen, Chi-Chen Yeh, Jin-Ching Lee

**Affiliations:** 1 Institute of Basic Medical Sciences, College of Medicine, National Cheng Kung University, Tainan, Taiwan; 2 Center of Infectious Disease and Signaling Research, College of Medicine, National Cheng Kung University, Tainan, Taiwan; 3 Doctoral Degree Program in Marine Biotechnology, National Sun Yat-Sen University, Kaohsiung, Taiwan; 4 Department of Biomedical Science and Environmental Biology, Cancer Center, Kaohsiung Medical University Hospital, Kaohsiung Medical University, Kaohsiung, Taiwan; 5 Department of Biotechnology, College of Life Science, Kaohsiung Medical University, Kaohsiung, Taiwan; 6 Department of Fragrance and Cosmetic Science, College of Pharmacy, Kaohsiung Medical University, Kaohsiung, Taiwan; 7 Graduate Institute of Medicine, College of Medicine, Kaohsiung Medical University, Kaohsiung, Taiwan; 8 Graduate Institute of Natural Products, College of Pharmacy, Kaohsiung Medical University, Kaohsiung, Taiwan; Yong Loo Lin School of Medicine, National University of Singapore, Singapore

## Abstract

In addition to the previous investigations of bioactivity of aqueous extract of the edible *Gracilaria tenuistipitata* (AEGT) against H_2_O_2_-induced DNA damage and hepatitis C virus replication, the purpose of this study is to evaluate the potential therapeutic properties of AEGT against inflammation and hepatotoxicity using lipopolysaccharide (LPS)-stimulated mouse RAW 264.7 cells, primary rat peritoneal macrophages and carbon tetrachloride (CCl_4_)-induced acute hepatitis model in rats. AEGT concentration-dependently inhibited the elevated RNA and protein levels of inducible nitric oxide synthase and cyclooxygenase-2, thereby reducing nitric oxide and prostaglandin E2 levels, respectively. Moreover, AEGT significantly suppressed the production of LPS-induced proinflammatory cytokines, including interleukin (IL)-1β, IL-6 and tumor necrosis factor-α. These inhibitory effects were associated with the suppression of nuclear factor-kappa B activation and mitogen-activated protein kinase phosphorylation by AEGT in LPS-stimulated cells. In addition, we highlighted the hepatoprotective and curative effects of AEGT in a rat model of CCl_4_-intoxicated acute liver injury, which was evident from reduction in the elevated serum aspartate aminotransferase and alanine aminotransferase levels as well as amelioration of histological damage by pre-treatment or post-treatment of AEGT. In conclusion, the results demonstrate that AEGT may serve as a potential supplement in the prevention or amelioration of inflammatory diseases.

## Introduction

Inflammation, considered an innate immune response beneficial to host survival, is a complex biological response of living organisms to harmful stimuli, such as infection, cellular damage, and tissue injury [1]. The inflammatory reaction includes a number of cellular and biochemical alterations involving the downstream regulation of proinflammatory protein expression and the upregulation of anti-inflammatory protein expression that facilitate the recruitment of immune cells, whereas pro-inflammatory cytokines facilitate this process [2], [3]. However, inappropriate control and a prolonged inflammatory response have been identified as crucial risk factors in the development of various chronic diseases such as autoimmune disorders, cancer, and vascular diseases [4]. Two important mediators of inflammation, inducible nitric oxide synthase (iNOS) and cyclooxygenase-2 (COX-2), regulate the inflammatory process by producing nitric oxide (NO) and prostaglandins (PG) E2 (PGE_2_), respectively [5]. Therefore, a compound with dual inhibitory effects on iNOS and COX-2 expression would have great potential in improving the treatment of chronic inflammation.

Lipopolysaccharide (LPS) is one of the major factors that stimulate the inflammatory response by stimulating various proinflammatory mediator cytokines such as interferon, interleukin-1 (IL-1β), interleukin-6 (IL-6), and tumor necrosis factor-α (TNF-α). In LPS-induced inflammation, the binding of LPS to the toll-like receptor 4 (TLR4)/CD14/MD2 complex stimulates the recruitment of both cytoplasmic MyD88 and TRIF adaptor proteins, which activates nuclear factor-kappa B (NF-κB) and mitogen-activated protein kinase (MPAK) signaling [6]. NF-κB signaling is an important mediator of the inflammatory response, cellular proliferation, and cell adhesion. NF-κB activation is controlled by the IκB kinase (IKK) complex, which induces IκB phosphorylation at two specific serine residues (Ser^32^ and Ser^36^), resulting in IκB degradation through the ubiquitin–proteasome system [2], [7]. Subsequently, the free NF-κB translocates to the nucleus and binds to specific binding sites in the promoter regions of its target genes, such as iNOS and COX-2 [8], [9]. The MAPK family consists of extracellular signal-regulated kinase (ERK), c-Jun N-terminal kinase (JNK), and p38 MAPK. Persistent activation of the MAPK signaling pathway has been revealed to increase the development of human inflammatory diseases due to the induction of iNOS expression [10]. Hence, targeting the NF-κB and MAPK signaling pathways is considered as an attractive therapeutic strategy for the development of anti-inflammatory drugs.

Liver diseases with severe hepatocyte damage caused by alcohol, viral infection or non-alcoholic steatohepatitis are highly associated with acute or chronic inflammation [11], [12]. Numerous type of cells, such as natural killer cells, T cells, dendritic cells and macrophages, are recruited during liver inflammation [11]. The hepatic resident macrophage play a critical role to excite liver injury because of great production of inflammatory cytokines including TNF-α, IL-1β and IL-6 and reactive oxygen species in response to inflammatory stimuli [11]. Administration of carbon tetrachloride (CCl_4_) to murine is a classical experimental model of severe liver injury involving in production of inflammatory cytokines and recruitment of inflammatory cells, leading to liver architectural damage and dysfunction [13], [14], [15].

Many different species of marine algae produce rich active substances that exhibit biological activity, and the substance are used in the treatment of inflammatory diseases [16], [17]. *Gracilaria* is a genus of red algae that is abundant in Taiwan. Recently, our studies revealed that the aqueous extract of *Gracilaria tenuistipitata* (AEGT) exerted protective effects against DNA damage and exhibited antioxidant activity [18]. In addition, AEGT inhibits hepatitis C virus (HCV) replication by regulating the expression of HCV-induced COX-2 and inhibits virus-induced inflammation *in vitro* [19]. In the present study, we evaluated the anti-inflammatory effect of AEGT using LPS-stimulated RAW264.7 and primary rat peritoneal macrophages, and found that AEGT exerted prophylactic and curative effects of anti-inflammatory activity by reducing iNOS, COX-2, TNF-α, IL-1β, and IL-6 expression. We further investigated the effect of AEGT on the NF-κB and MAPK signaling pathways to clarify its inhibitory mechanism. Finally, the hepatoprotective and anti-inflammatory effects of AEGT was examined in an animal model. These results provide evidence that AEGT may be a potential anti-inflammatory supplement.

## Materials and Methods

### Experimental Animals and Ethics Statement

Male wistar rats were used in this study and the rats were obtained from BioLasco Taiwan Co. Ltd. The Animal Research Committee of Kaohsiung Medical University of Taiwan ratified the experimental protocol and approved this study (Permit Number: 101014). All rats received humane care and were fed with standard rodent chew and water ad libitum. All surgery was performed under zoletil 50 anesthesia, and all efforts were made to minimize suffering. Rats were acclimatized under a standard laboratory condition following the Animal Use Protocol of Kaohsiung Medical University for a week before experiment. *G. tenuistipitata* is not an endangered or protected seaweed. *G. tenuistipitata* was collected in the No.129, Kouhu village, Kouhu Township, Yunlin County 653, Taiwan (R.O.C.), which is not a protected area but is privately owned by SHUI-TUI LI Taiwan. Mr. LI permits research in their reserves and no specific permits are required for field studies like the one described here.

### Reagents

Lipopolysaccharide (LPS) and Paclitaxel (Taxol) were obtained from Sigma Chemicals Inc. and stored as 1 mg/ml and 10 mM, respectively. The Griess reagents were obtained from Promega Corporation, Madison, WI.

### Preparation of AEGT

Aqueous extract of *G. tenuistipitata* (AEGT) was prepared as before described [19]. The major constituents contain polyphenol, favonoid, and ascorbic acid. The amount of polyphenol, flavonoid, and ascorbic acid were respectively recorded as 98.94±2.43 µg, 22.59±1.08 µg, and 1.59±0.18 µg in 1 mg of dry extract.

### Analysis of AEGT Extract by NMR Method

The extract was dissolved in D_2_O and then NMR spectrum was recorded on a Varian Unity-plus 400 MHz FT-NMR. The chemical shift values are reported in parts per million (δ) using D_2_O as an internal standard.

### Cell Culture

The RAW264.7 murine macrophage cells were cultured in Dulbecco’s modified Eagle’s medium (DMEM) with 10% fetal bovine serum (FBS), 1% non-essential amino acids, and 1% antibiotic-antimycotic in 5% CO_2_ supplement at 37°C.

### Isolation of Peritoneal Macrophages

Male Wistar rats (150–200 g) obtained from BioLasco Taiwan Co. Ltd. were maintained for 2 weeks in humane care and were fed with standard rodent chew and water ad libitum facility. Wistar rats were anesthetized by an intramuscular injection of Zoletil 50 (75 mg/kg). Peritoneal macrophages were obtained by sterile lavage of the peritoneal cavity with cold DMEM medium. The lavage fluid was centrifuged at 1,000 g for 10 min and the supernatant was decanted and the cells were resuspended in DMEM with 10% fetal bovine serum (FBS), 1% non-essential amino acids, and 1% antibiotic-antimycotic, followed by plating in a 24-well plates at 37°C [20].

### Cell Viability Assay

RAW264.7 cells and primary rat peritoneal macrophages were seeded in a 96-well plate at density of 2.5×10^4^ and 10^5^ cells, respectively. Cells were treated with 400 or 800 µg/ml of AEGT for 1 hour and then stimulated with 1 µg/ml of LPS for 24 h. The cell viability was analyzed by CellTiter 96 AQ_ueous_ One Solution Cell Proliferation Assay (Promega) after 24 h treatment. Furthermore, flow cytometric analysis using Annexin-V/propidium iodide was performed to clearly verify the cell viability. Treatment of 1 µM Taxol served as a positive control in these cell viability assays.

### Western Blotting

Western blotting was performed as previously described. Anti-COX-2 antibody was obtained from Cayman, Ann Arbor, MI. Anti-GAPDH and anti-lamin B antibodies were obtained from GeneTex. Anti-phosphorylated IκBα, IKKα/β, p38, JNK, ERK and anti-total iNOS, IκBα, IKKα, IKKβ, p65, p38, JNK, ERK antibodies were obtained from Cell Signaling (Beverly, MA, USA).

### Quantification of RNA Level

Quantitative real-time reverse-transcription polymerase chain reaction (qRT-PCR) was performed as previously described [19]. Relative mRNA levels were normalized by endogenous cellular gene glyceraldehydes-3-phosphoate dehydrogenase (*gapdh*). Primers used in the study are listed in Table 1.

**Table 1:** Oligonucleotide sequences for real-time RT-PCR.

Oligonucleotide Name	Sequence 5′–3′
5′ iNOS	5′- CTTTGGTGCTGTATTTCC
3′ iNOS	5′- TGTGACCTCAGATAATGC
5′ COX-2	5′- CCGAGGTGTATGTATGAG
3′ COX-2	5′- TGGGTAAGTATGTAGTGC
5′ GAPDH	5′- GTCTTCACCACCATGGAGAA
3′ GAPDH	5′- ATGGCATGGACTGTGGTCAT
5′ TNF-α	5′- CCTGTGAGGAGGACGAAC
3′ TNF-α	5′- AAGTGGTGGTCTTGTTGC
5′ IL-1β	5′- GGAGAATGACCTGAGCAC
3′ IL-1β	5′- GACCAGACATCACCAAGC
5′ IL-6	5′- TCAGAATTGCCATTGCACA
3′ IL-6	5′- GTCGGAGGCTTAATTACACATG

### Measurement of Nitrite and PGE_2_ in Culture Media

The RAW264.7 cells were seeded in a 24-well plate and treated with 400 and 800 µg/ml of AEGT for 1 hour and then stimulated with 1 µg/ml of LPS for 24 h. The supernatants were collected to analyze nitrite and PGE_2_ levels. Nitrite level was analyzed by Griess reagent (Promega) according to the manufacturer’s instructions. PGE_2_ level was analyzed by an ELISA kit (Biotrak, Amersham Bioscience) according to the manufacturer’s instructions.

### Measurement of Pro-inflammatory Cytokines in Culture Media

The RAW264.7 cells and primary rat peritoneal macrophages were seeded in a 24-well plate and treated with 400 and 800 µg/ml of AEGT for 1 hour and then incubated with 1 µg/ml of LPS for 24 hours. The culture media was collected and the TNF-α, IL-1β and IL-6 levels were analyzed by ELISA kits (Invitrogen, Carlsbad, CA, USA).

### NF-κB Reporter Assay

NFκB reporter assay was performed as previously described [21]. Briefly, the RAW264.7 cells were seeded in a 24-well plate and the reporter plasmid pNFκB-Luc and pCMV-SEAP were co-transfected into cells. After 24 h, the transfected cells were treated with 400 or 800 µg/ml of AEGT for 1 h. After incubation with LPS (1 µg/ml) for 2 h, the cells were collected, and the luciferase and SEAP activity were analyzed according to the protocol of the Steady-Glo Luciferase Assay System (Promega, Madison, WI) and Phospha-Light assay kit (Tropix, Foster City, CA), respectively. SEAP activity served as an internal control.

### Nuclear Fraction Preparation

The RAW264.7 cells were seeded in a 10-cm dish and then treated with 400 or 800 µg/ml of AEGT for 1 h. After incubation with LPS (1 µg/ml) for 2 h, the cells were collected for nuclear fraction prepared. The nuclear fraction was prepared as previously described [19].

### Hepatoprotective Activity Assay *in vivo*

Carbon tetrachloride (CCl_4_) was used to induce acute hepatotoxicity as previously described [22]. Wistar rats weighing 180–200 g were randomly divided into 4 groups (n = 5/group): Group 1: saline, group 2: saline+CCl_4_, group 3∶150 mg/kg of AEGT+CCl_4_, and group 4∶300 mg/kg of AEGT+CCl_4_. Rats were given saline or different doses of AEGT by oral gavage for 4 days once a day. On day 4, group 1 received corn oil (2.5 ml/kg) and group 2–4 received 20% CCl_4_ in corn oil (2.5 ml/kg) by intraperitoneal injection (i.p.) post 1 h of saline or AEGT treatment. After 24 h, the rats were sacrificed by CO_2_ asphyxiation. Blood samples were collected as previously described [22]. Briefly, the blood samples were collected by cardiac puncture and centrifuged at 3,000 rpm for 10 minutes to separate serum. The serum was used to analyze liver function by determining aspartate aminotransferase (AST) and alanine aminotransferase (ALT) with an autoanalyzer (Hitachi 7050, Tokyo, Japan).

### Histopathology

The histopathological observation was also performed as previously described [22]. The liver tissues were collected and stained with hematoxylin and eosin to observe the liver injury by photomicroscope.

### Analysis of Inflammatory Protein Expression and Secretion *in vivo*

CCl_4_ was used to induce acute hepatotoxicity as previously described [22]. Wistar rats weighing 180–200 g were randomly divided into 4 groups (n = 5/group): Group 1: saline, group 2: saline+CCl_4_, group 3∶150 mg/kg of AEGT+CCl_4_, and group 4∶300 mg/kg of AEGT+CCl_4_. Rats were given saline or different doses of AEGT by oral gavage for 4 days once a day. On day 4, group 1 received corn oil (2.5 ml/kg) and group 2–4 received 20% CCl_4_ in corn oil (2.5 ml/kg) by intraperitoneal injection (i.p.) post 1 h of saline or AEGT treatment. After 24 h, rats were sacrificed by CO_2_ asphyxiation. Blood samples were collected as previously described [22]. Blood was collected and centrifuged at 3,000 rpm for 10 min to separate the serum for determining TNF-α, IL-1β and IL-6 levels by ELISA kits (Invitrogen, Carlsbad, CA, USA). In addition, the liver tissue were collected and protein were extracted for western blotting with anti-COX-2 (Cayman, Ann Arbor, MI.), anti-iNOS or anti-GAPDH (abcam) antibody.

### Statistical Analysis

The data were expressed as mean ± SD of at least three independent experiments. Statistical calculations were analysed by the Student’s *t*-test; *p*-values <0.01 were considered statistically significant.

## Results

### Effect of AEGT on Cell Viability

**Figure 1:**
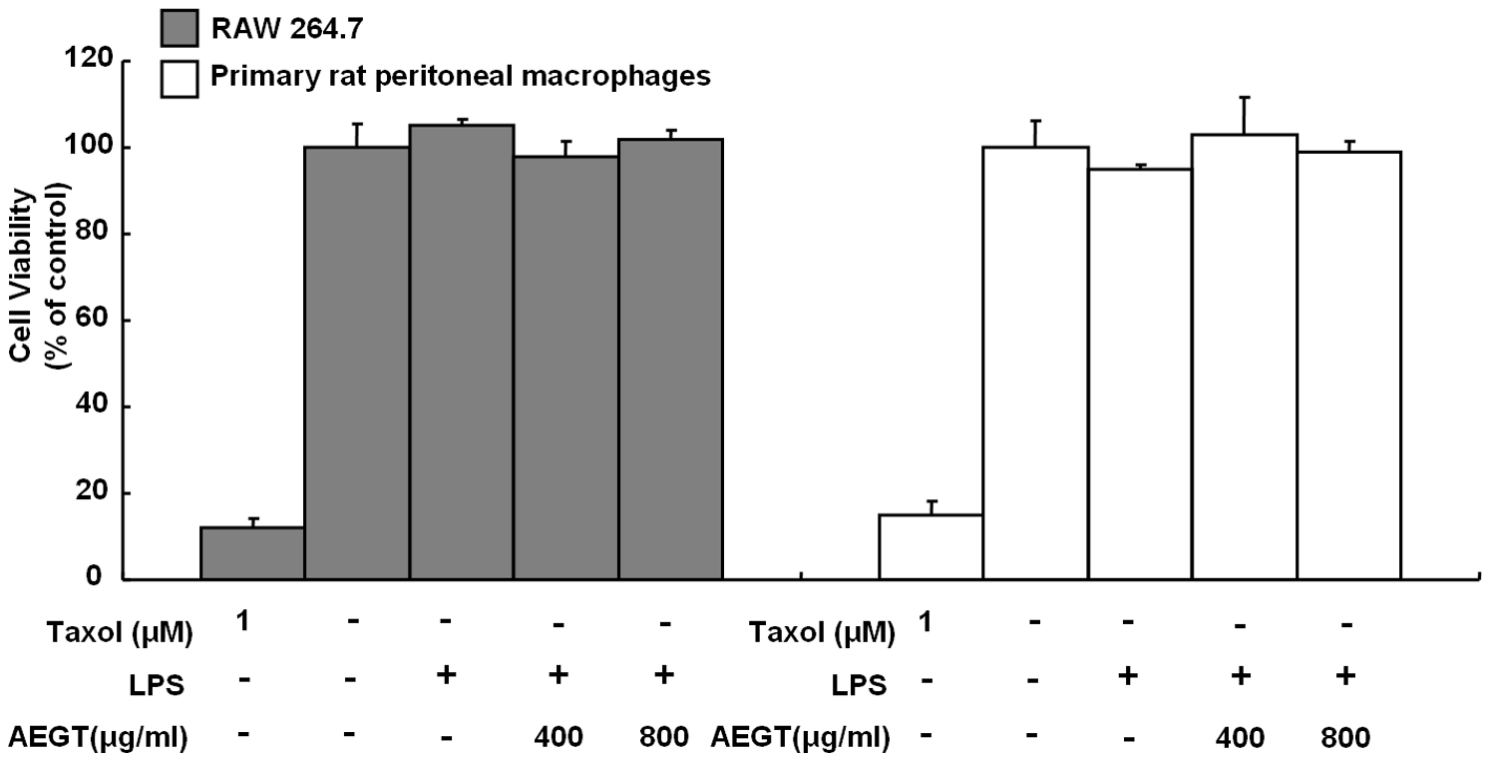
Effect of AEGT on the cell viability of RAW 264.7 cells and primary rat peritoneal macrophages. Cells were seeded in a 96-well plate and treated with 400 or 800 µg/ml AEGT for 1 h, and then incubated with 1 µg/ml LPS for 24 hours. Cell viability was measured by the MTA assay. Treatment of 1 µM Taxol served as a positive control. Error bars indicate the means ± SD of three independent experiments. *P<0.05; **P<0.01.

To detect the cytotoxicity of AEGT in RAW 264.7 cells and primary rat peritoneal macrophages, the cells were incubated with 400 or 800 µg/ml AEGT for 1 h and then incubated with 1 µg/ml LPS for 24 h, after which the MTS assay and flow cytometric analysis using Annexin-V/propidium iodide staining were performed. As shown in Fig. 1 and Fig. S2, the viability of RAW 264.7 cells and primary rat peritoneal macrophages were not significantly affected by treatment with AEGT up to 800 µg/ml compared with that of the cells receiving no LPS, AEGT or Taxol treatment.

### Effects of AEGT on LPS-induced iNOS/COX-2 Expression and NO/PGE_2_ Production

**Figure 2:**
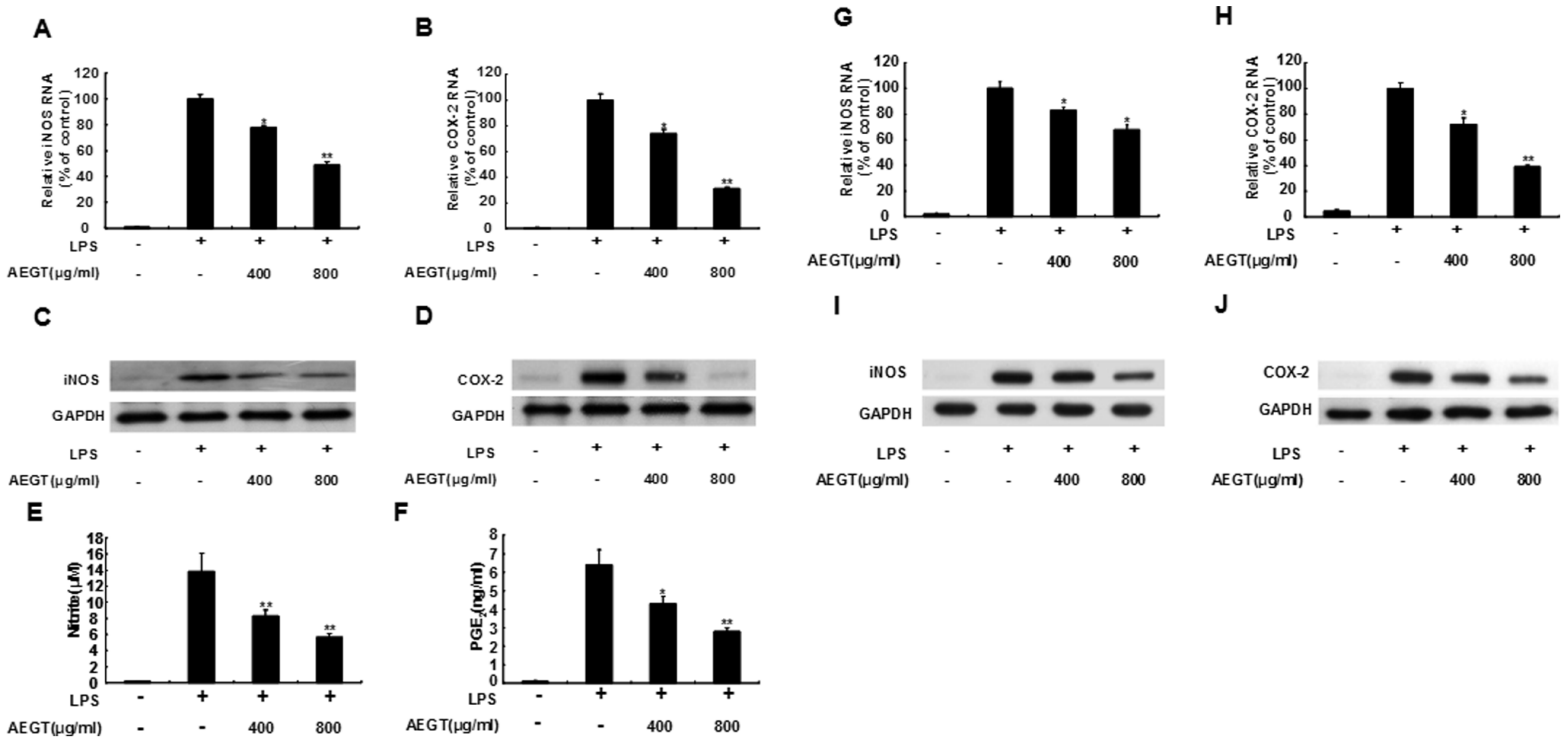
Effect of AEGT on NO/PGE_2_ production and iNOS/COX-2 expression in LPS-stimulated RAW 264.7 cells (A–E) and primary rat peritoneal macrophages (G–J). Cells were seeded in a 24-well plate, treated with 400 or 800 µg/ml AEGT for 1 h, and then incubated with 1 µg/ml LPS for 24 h. The cell lysates were collected to determine the RNA (A, B, G and H) and protein (C, D, I and J) levels of iNOS and COX-2 by qRT-PCR with specific primers and western blot analysis with antibodies against iNOS and COX-2, respectively. The efficiency of inhibition was determined as the percent RNA levels relative to those in the cells treated with LPS alone. The supernatant was collected to quantify NO (E) and PGE_2_ (F) levels using Griess reagents and an ELISA kit, respectively. Error bars indicate the means ± SD of three independent experiments. *P<0.05; **P<0.01.

To evaluate the inhibitory effect of AEGT on the proinflammatory mediators NO and PGE_2_, we first measured the gene expression of iNOS and COX-2. The RAW 264.7 cells were treated with 400 or 800 µg/ml AEGT for 1 h and then incubated with 1 µg/ml LPS for 24 h. The RNA and protein levels of iNOS and COX-2 were measured by qRT-PCR and western blot analysis, respectively. As shown in Fig. 2, AEGT treatment dramatically reduced the RNA (Fig. 2A and 2B) and protein (Fig. 2C and 2D) levels of iNOS and COX-2 in a concentration-dependent manner compared with LPS stimulation without AEGT pretreatment. We next detected NO and PGE_2_ levels using Griess reagents and an ELISA kit under the same experimental condition. The results indicated that 800 µg/ml AEGT decreased NO and PGE_2_ production to 6.3±0.8 µM and 2.8±0.5 ng/ml, respectively, compared to LPS which stimulated NO and PGE_2_ production to 14.2±2.2 µM and 6.6±0.9 ng/ml, respectively (Fig. 2E and 2F). Subsequently, we employed the primary rat peritoneal macrophage cell culture to further evaluate the inhibitory effect of AEGT on LPS-stimulated inflammation *exo vivo* under the same experimental conditions. Consistent with the *in vitro* data obtained in LPS-treated RAW 264.7 cells, AEGT significantly reduced LPS-stimulated RNA (Fig. 2G and 2H) and protein (Fig. 2I and 2J) levels of iNOS and COX-2 in a concentration-dependent manner.

### Effects of AEGT on LPS-induced Pro-inflammatory Cytokines Production

**Figure 3:**
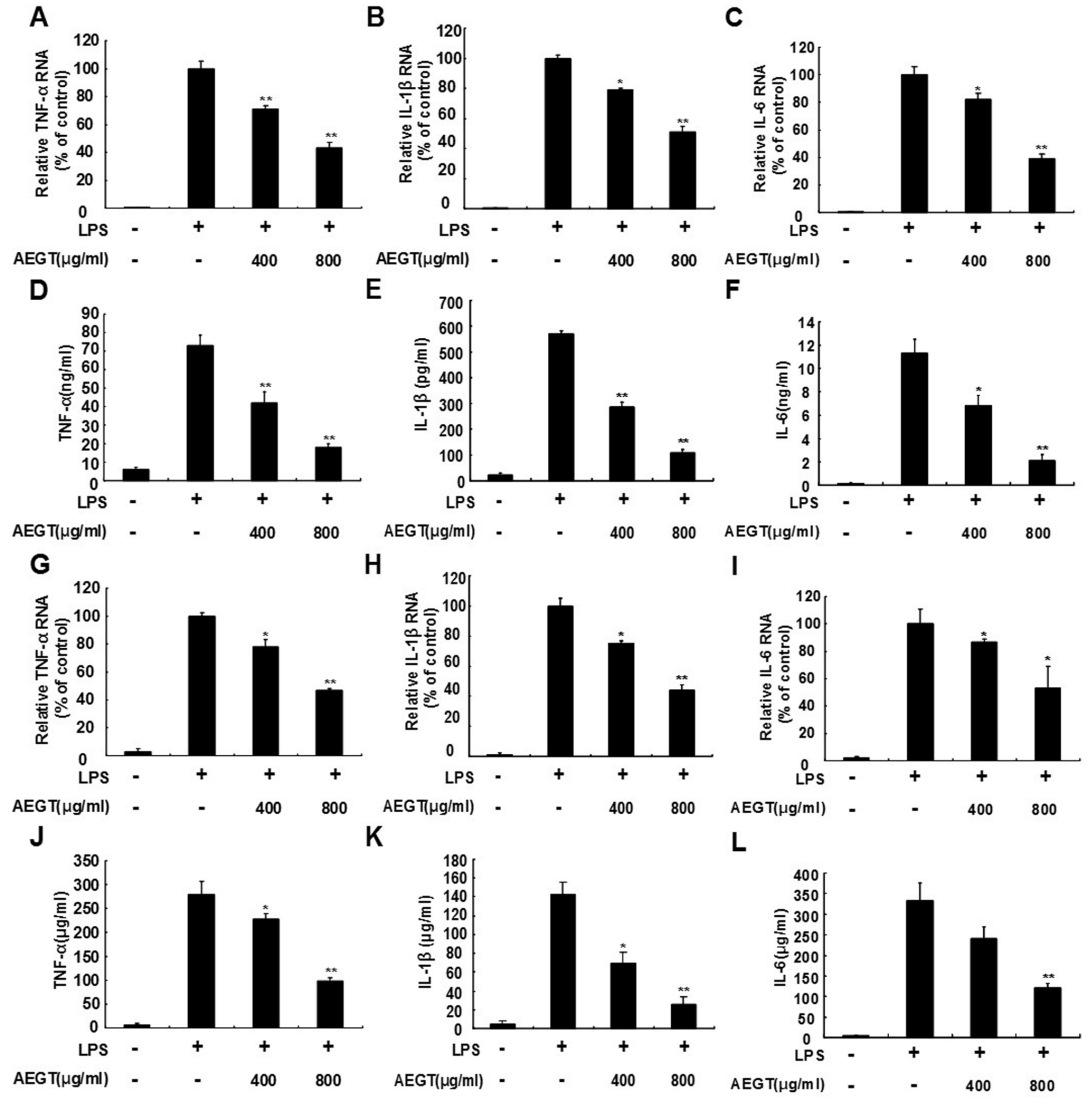
Effect of AEGT on the production and gene expression of proinflammatory cytokines in LPS-stimulated RAW 264.7 cells (A–F) and primary rat peritoneal macrophages (G–L). Cells were seeded in a 24-well plate treated with 400 or 800 µg/ml AEGT for 1 h, and then incubated with 1 µg/ml LPS for 24 h. The RNA (A–C and G–I and secreted protein levels of proinflammatory cytokines including TNF-α (D and J), IL1-β (E and K) and IL6 (F and L), were analyzed by qRT-PCR and ELISA, respectively. The efficiency of inhibition was determined as the percent RNA levels relative to those in the cells treated with LPS alone. Error bars indicate the means ± SD of three independent experiments. *P<0.05; **P<0.01.

Many proinflammatory cytokines, including TNF-α, IL-1β and IL-6, are suggested to be crucial mediators of the development of inflammatory diseases [23], [24]. To investigate the effect of AEGT on proinflammatory gene expression, the RAW 264.7 cells were treated with 400 or 800 µg/ml AEGT for 1 h and then incubated with 1 µg/ml LPS for 24 h. qRT-PCR demonstrated that LPS-induced upregulation of TNF-α, IL-1β, and IL-6 RNA levels was significantly decreased in the AEGT-treated cells in a concentration-dependent manner compared with the AEGT-untreated cells in the absence or presence of LPS (Fig. 3A–3C). To further investigate the effect of AEGT on proinflammatory cytokine production, we detected TNF-α, IL-1β, and IL-6 levels by ELISA. As shown in Fig. 3D–3F, AEGT significantly decreased LPS-induced TNF-α, IL-1β, and IL-6 production in RAW 264.7 cells by approximately 3-fold to 5-fold over control, respectively, at concentration of 800 µg/ml. In addition, we used an *ex vivo* primary rat peritoneal macrophage cell culture to confirm the inhibitory effect of AEGT on LPS-elevated RNA levels (3G–3I) and protein production (3J–3L) of proinflammatory cytokines under the same experimental conditions.

### Effects of AEGT on LPS-induced IKK/IκB/NFκB Signaling Pathway

**Figure 4:**
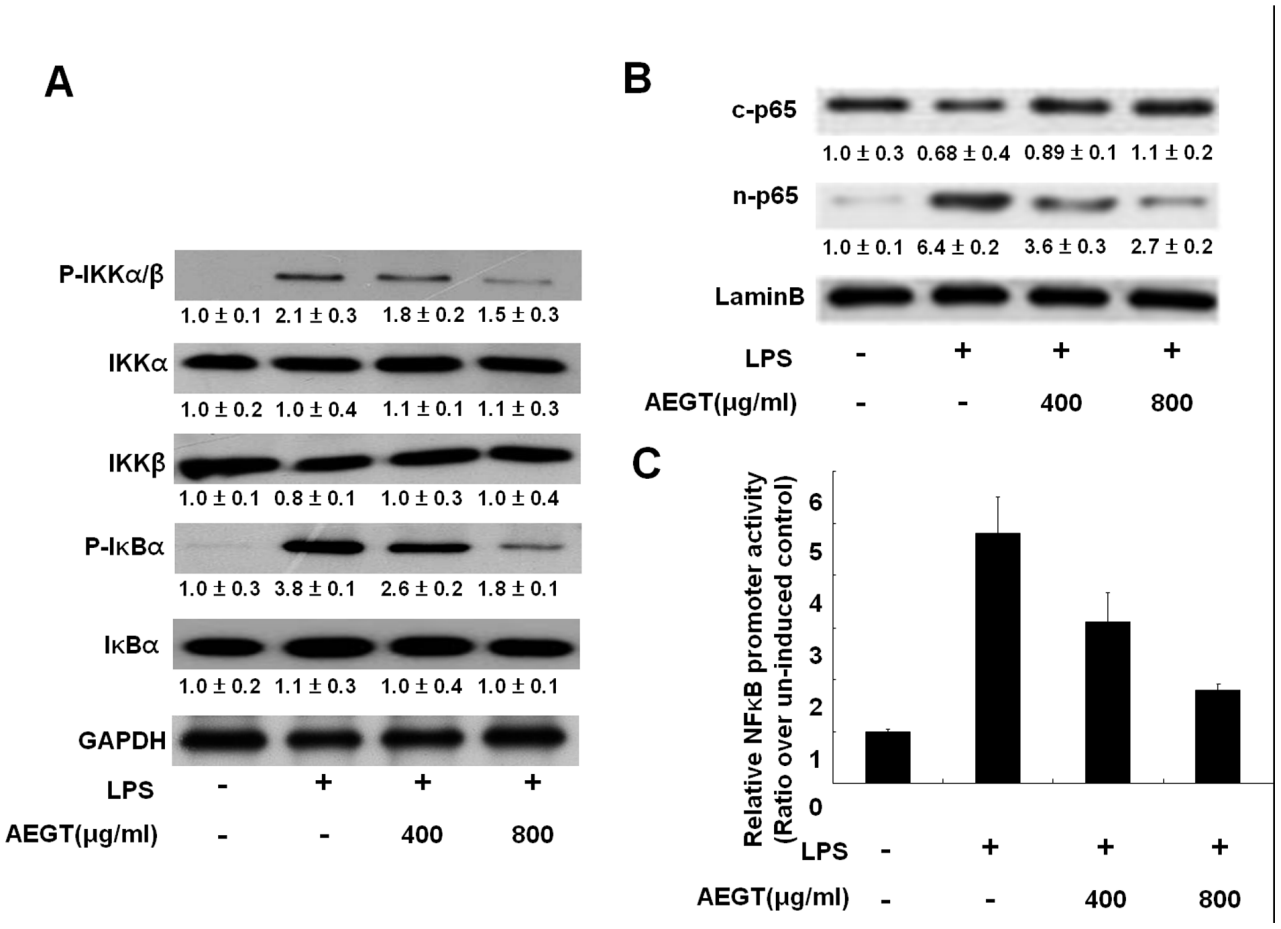
Effect of AEGT on NF-κB activation in LPS-stimulated RAW 264.7 cells. (A) The RAW264.7 cells were seeded in a 24-well plate, and treated with 400 or 800 µg/ml AEGT for 1 h and then incubated with 1 µg/ml LPS for 2 h. The levels of total protein and phosphorylated IKKα, IKKβ, and IκBα were analyzed by western blot analysis with specific antibodies. The relative value of the LPS- group or AEGT/LPS-treated group over the untreated group was measured by densitometry following normalization to cellular GAPDH levels. In the NF-κB activation assay, the cells were transfected with a pNFκB-Luc reporter plasmid. Subsequently, the transfected cells were treated with 400 or 800 µg/ml AEGT for 1 h, and then incubated with 1 µg/ml LPS for 2 h. The cell lysates and nuclear fraction were collected for western blot (B) and luciferase activity (C) analyses, respectively. The basal level of promoter activity in the AEGT/LPS-untreated cells was defined as 1. Error bars indicate the means ± SD of three independent experiments. *P<0.05; **P<0.01.

The IKK/IκB/NF-κB signaling pathway has been reported to regulate many of the genes involved in the inflammatory response and production of inflammatory cytokines and proinflammatory enzymes [25], [26], [27]. To investigate whether the inhibitory effects of AEGT on the production of proinflammatory mediators (iNOS and COX-2) and proinflammatory cytokines (TNF-α, IL-1β, and IL-6) are associated with the blockage of the IKK/IκB/NF-κB signaling pathway, we first investigated the effects of AEGT on IKKα/β kinase and IκBα phosphorylation using western blot analysis. The RAW 264.7 cells were pretreated with AEGT at increasing concentrations for 1 h and then treated with LPS for 2 h. As shown in Fig. 4A, LPS-induced phosphorylation of IKKα/β and IκBα was significantly decreased in the AEGT (800 µg/ml)-treated cells compared with the AEGT-untreated cells in the absence or presence of LPS. We next investigated the effect of AEGT on the nuclear translocation of NF-κB subunit p65 and NF-κB-mediated promoter activity. The RAW 264.7 cells were transiently transfected with the *cis*-reporting plasmid pNFκB-Luc and then incubated with AEGT and LPS under the same experimental conditions. As shown in Fig. 4B, western blot analysis demonstrated that the nuclear fraction of p65 was reduced by AEGT pretreatment. As expected, NF-κB-mediated luciferase activity was significantly inhibited by AEGT in a concentration-dependent manner (Fig. 4C). The data suggest that AEGT eliminates the LPS-induced proinflammatory mediators (iNOS and COX-2) and proinflammatory cytokines (TNF-α, IL-1β and IL-6) by inhibiting IKK/IκB/NF-κB signaling.

### Effect of AEGT on LPS-induced MAPK Activation

**Figure 5:**
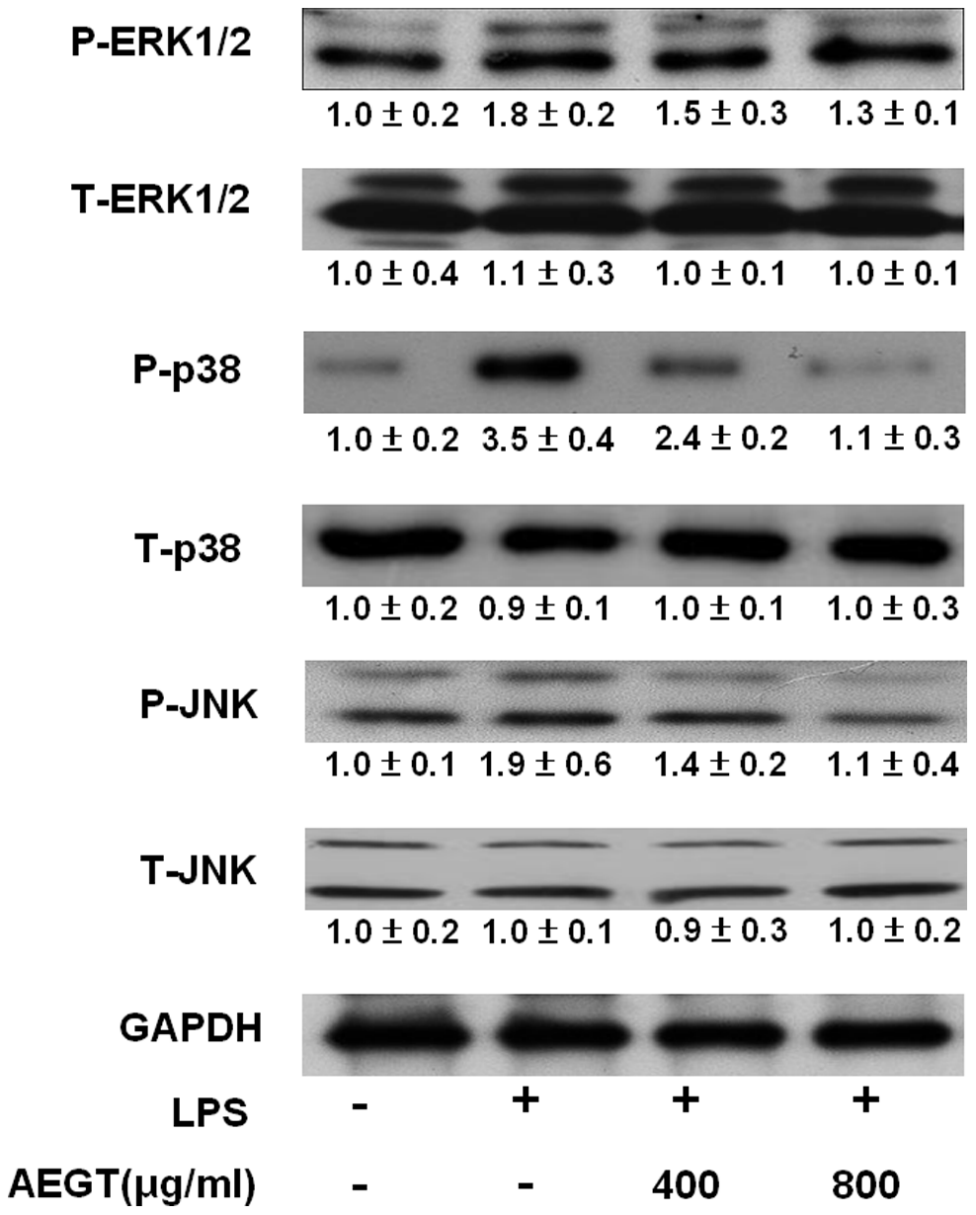
Effect of AEGT on MAP kinase phosphorylation in LPS-stimulated RAW 264.7 cells. The RAW264.7 cells were seeded in a 24-well plate, treated with 400 or 800 µg/ml AEGT for 1 h and stimulated with 1 µg/ml LPS for 24 h. The cell lysates were collected, and p38, JNK, and ERK1/2 phosphorylation was analyzed by western blot analysis with specific antibodies. The relative value of the LPS- group or AEGT/LPS-treated group over the untreated group was measured by densitometry following normalization to cellular GAPDH levels. The basal level of promoter activity in the AEGT/LPS-untreated cells was defined as 1. Error bars indicate the means ± SD of three independent experiments. *P<0.05; **P<0.01.

MAPK phosphorylation plays a pivotal role in the regulation of LPS-induced inflammatory mediators [28]. Hence, we performed western blot analysis to determine whether the suppression of proinflammatory mediators by AEGT was regulated through the MAPK pathway. The RAW 264.7 cells were pretreated with AEGT for 1 h followed by LPS treatment for 2 h, and the cell lysates were subjected to western blot analysis. As shown in Fig. 5, LPS significantly elevated JNK, ERK and p38 MAPK phosphorylation, whereas AEGT pretreatment inhibited the LPS-induced phosphorylation of JNK, ERK, and p38 MAPK. The results clarify that JNK, ERK and p38 MAPK inhibition by AEGT may contribute to anti-inflammatory activity in LPS-stimulated RAW 264.7 cells.

### Anti-inflammatory Effect of AEGT on Carbon Tetrachloride (CCl_4_)-induced Liver Injury in Rats

**Figure 6:**
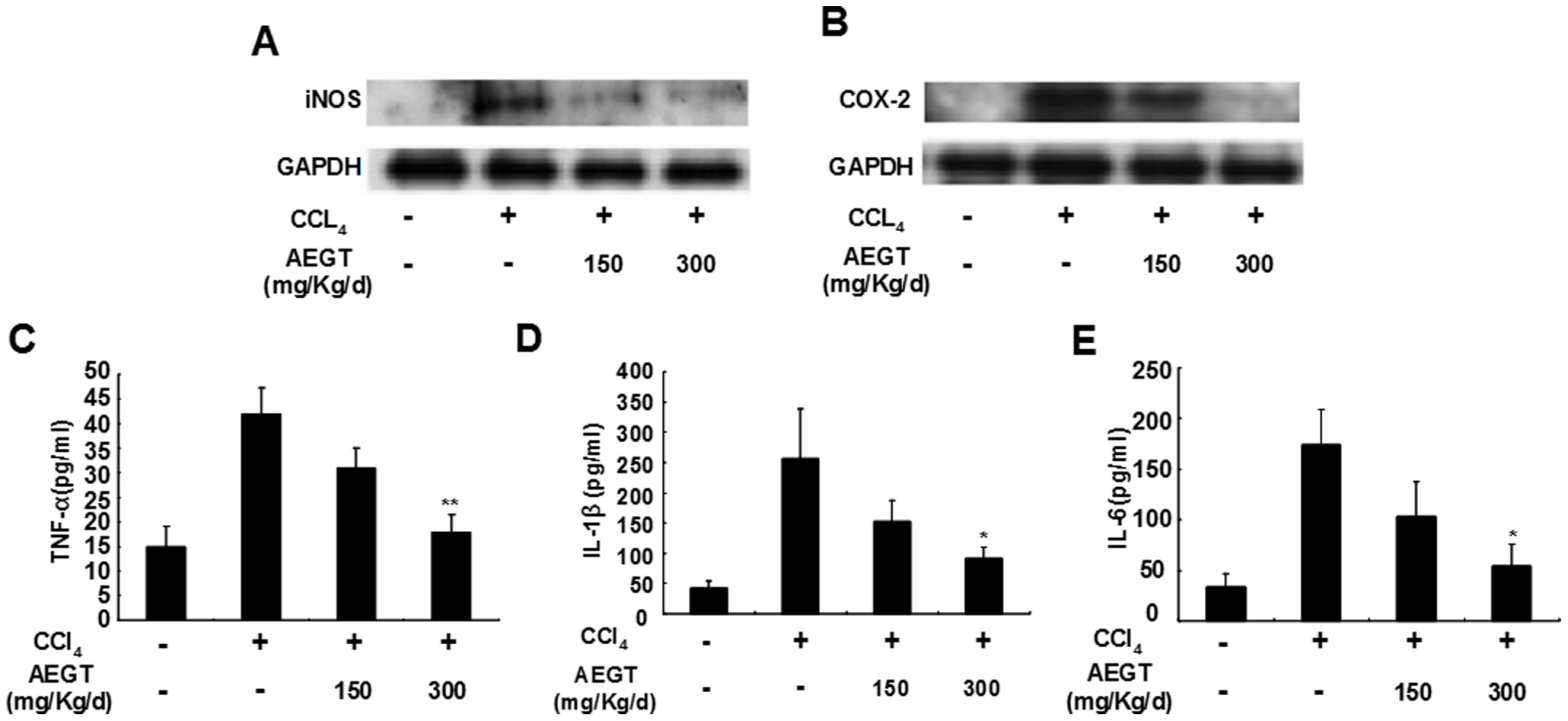
Effect of AEGT on CCl_4_-induced acute liver inflammation in Wistar rat. Rats were given the indicated doses of AEGT for 3 days and then administered CCl_4_ treatment for 1 day. Subsequently, rats were sacrificed to collect blood samples and liver sections for inflammatory parameter analysis of iNOS (A) and COX-2 (B) protein expression and secreted protein levels of proinflammatory cytokines including TNF-α (C), IL1-β (D) and IL6 (E). Values are presented as the means of five independent experiments. Error bars indicate the means ± SD of three independent experiments. *P<0.05; **P<0.01.

We next performed a rat model of CCl_4_-induced liver injury to evaluate the anti-inflammatory effect of AEGT *in vivo*. As shown in Fig. 6A and 6B, the CCl_4_-elevated protein levels of iNOS and COX-2 were markedly reduced by AEGT pre-treatment, compared with the saline-treated controls. Moreover, the levels of pro-inflammatory cytokines, including TNF-α, IL-1β and IL-6, in serum were measured by ELISA. Upon CCl_4_ stimulation, the protein levels of TNF-α, IL-1β and IL-6 in serum were increased by approximately 3, 5 and 4-fold, respectively, compared with the saline-treated controls. However, AEGT pre-treatment resulted in significant reduction of the protein levels of those of pro-inflammatory cytokines in a concentration-dependent manner (Fig. 6C–6E). These findings are consistent with the *in vitro* or *ex vivo* data observed above in RAW 264.7 cells or primary rat peritoneal macrophages treated with LPS.

### Hepatoprotective Effect of AEGT on Carbon Tetrachloride (CCl_4_)-induced Liver Injury in Rats

**Figure 7:**
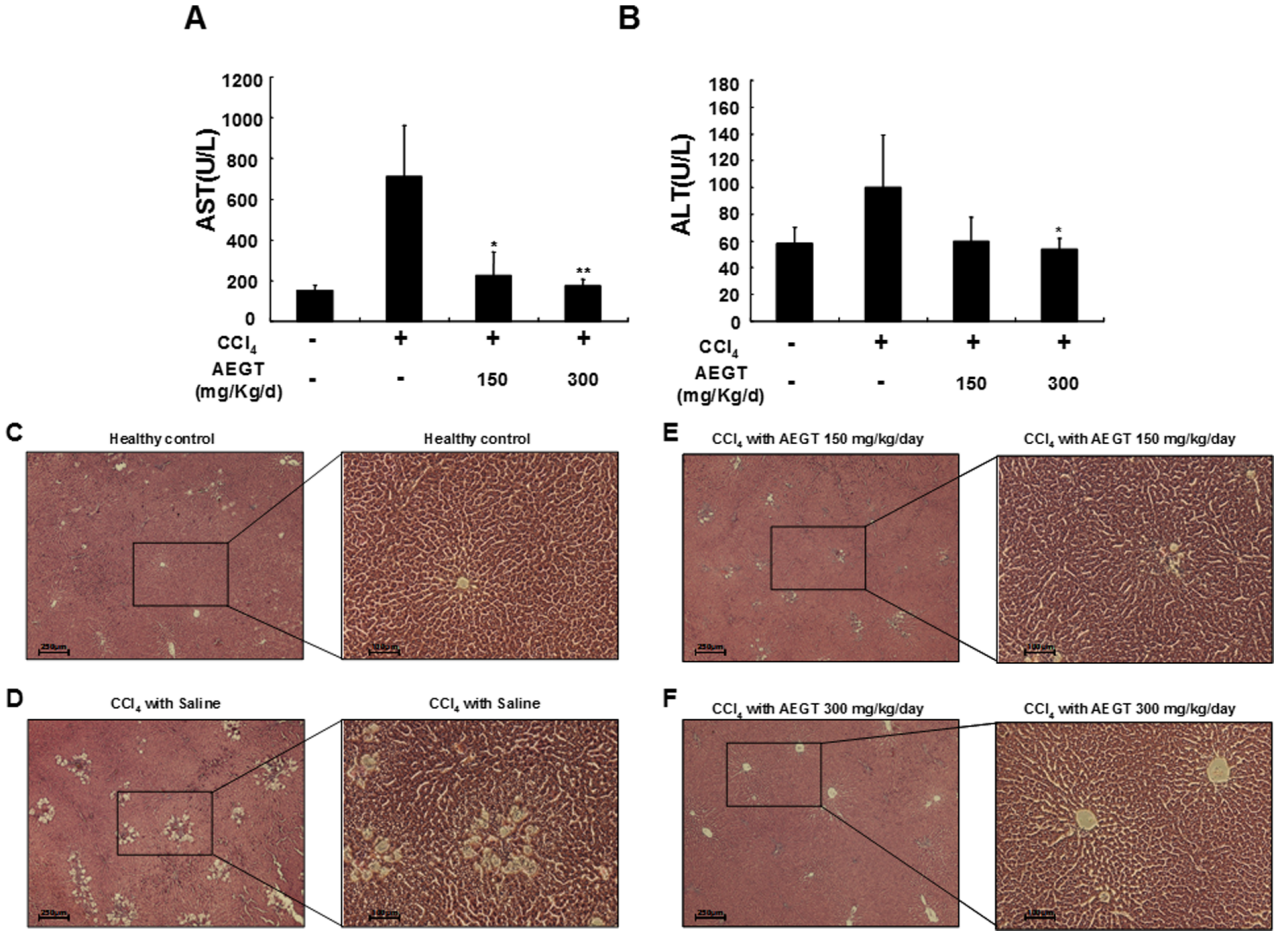
Effect of AEGT on CCl4-induced acute liver injury in Wistar rat. The Wistar rats were given the indicated doses of AEGT for 3 days and then administered CCl4 for 1 day. The rats were sacrificed to collect blood samples and liver sections for biochemical parameter analysis of (A) AST and (B) ALT and histopathological examination [(C) Healthy control, (D) CCl4 with saline, (E) CCl4 with 150 mg/kg/day AEGT, and (F) CCl4 with 300 mg/kg/day AEGT]. Values are presented as the means of five independent experiments. Error bars indicate the means ± SD of three independent experiments. *P<0.05; **P<0.01.

We generated a rat model of CCl_4_-induced liver injury to investigate the hepatoprotective effect of AEGT *in vivo*. As shown in Fig. 7A and 7B, the CCl_4_-induced AST and ALT protein levels were significantly decreased by AEGT pre-treatment compared with their levels after saline treatment. Furthermore, histopathological analysis demonstrated that the liver sections of the CCl_4_-intoxicated group displayed destructive hepatic cell necrosis, including ballooning degeneration and fatty change compared with the saline-treated control groups (Fig. 7C and 7D). In contrast with the AEGT-untreated groups, the AEGT-pretreated groups exhibited a regular cellular architecture with a central vein and no necrosis, inflammation, or vascular degeneration (Fig. 7E and 7F).

### Characterization of AEGT Extract

To realize the ingredients in AEGT, we analyzed the extract with a nuclear magnetic resonance (NMR). The spectrum was shown in supplementary data (Fig. S1). We found that the ingredients of crude extract were too complicated to identify in detail. In comparison with the database of our laboratory, we can primarily confirmed that the major component of is polysaccharide complex, designed as region I, and minor is phenoids molecules, designed as region II, according to the signal of chemical shift.

## Discussion

**Figure 8:**
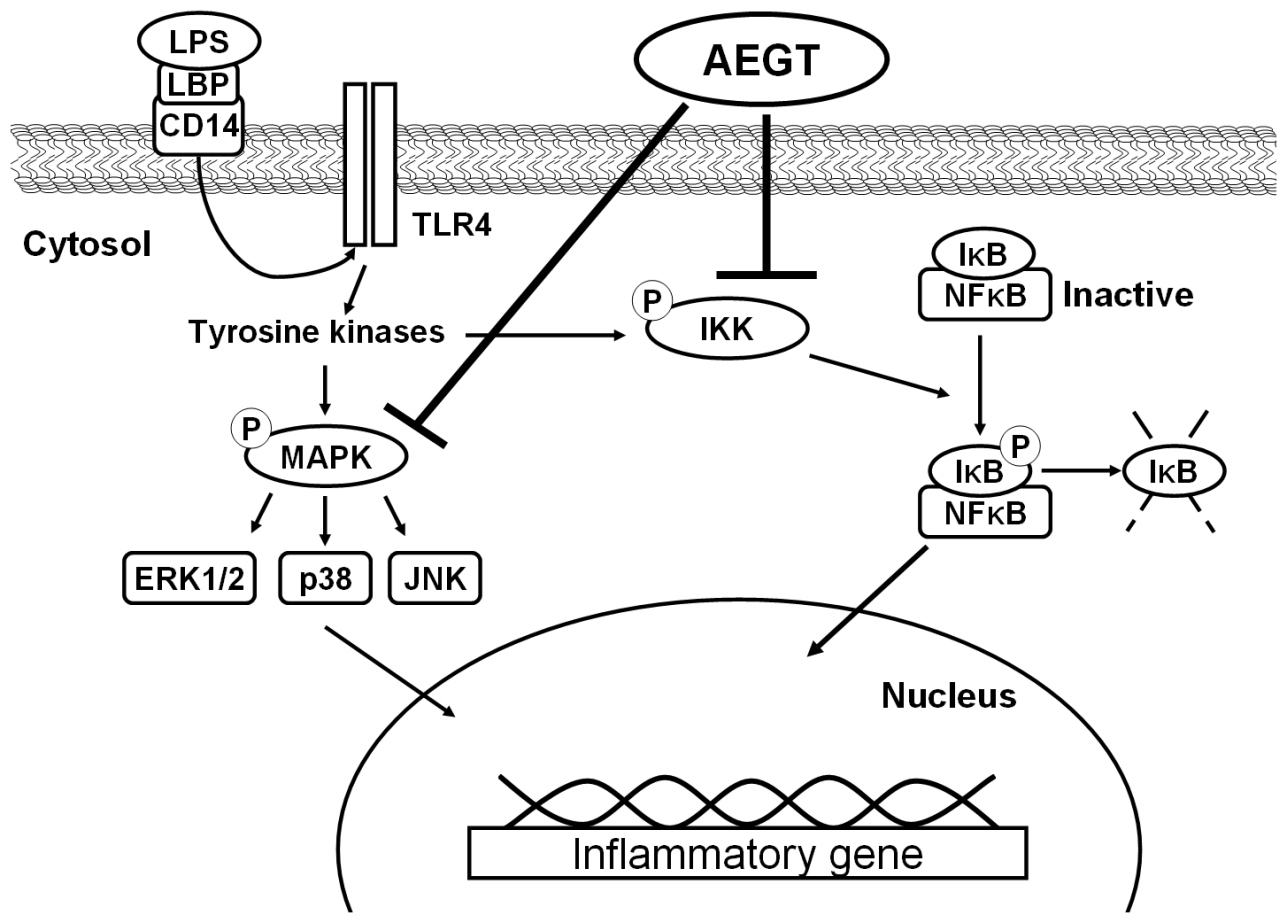
A proposed model for the inhibitory action of AEGT on LPS-induced inflammation. AEGT inhibits IKKα/β and MAPKs phosphorylation, eventually blocking NF-κB activation and proinflammatory gene expression.

Targeting COX-2 and iNOS has been considered an effective strategy to prevent inflammation diseases [29]. In this study, we revealed that AEGT efficiently downregulated LPS and CCl_4_-induced COX-2 and iNOS protein expression *in vitro*, *ex vivo* and *in vivo* rat models, respectively (Fig. 2 and Fig. 6). Several proinflammatory cytokines, such as TNF-α, IL-1β, and IL-6, are known to play an important role in stimulating iNOS and COX-2 in macrophages [25], [26], [27]. Our results demonstrated that AEGT remarkably inhibited the stimulation of TNF-α, IL-1β, and IL-6 by LPS at the transcriptional level (Fig. 3), indicating that the reduction in iNOS and COX-2 expression by AEGT may be associated with the blockage of these proinflammatory cytokines. NF-κB signaling, which regulates a number of inflammatory genes, including iNOS, COX-2, TNF-α, IL-1β, and IL-6, can be stimulated by pathological stimuli, such as viral infection, UV radiation, and free radicals, and its dysregulation is involved in the pathogenesis of many inflammatory disorders [30]. The results of this study revealed that AEGT treatment blocked IKKα/β-mediated IκBα phosphorylation, leading to the significant attenuation of NF-κB activation in response to LPS (Fig. 4). This action may contribute to the reduction in NO, PGE_2,_ and pro-inflammatory cytokine production under LPS-induced inflammation. MAPKs have been reported to play an important role in the regulation of LPS-induced inflammation by controlling NF-κB activation [31]. Our data demonstrated that AEGT JNK, ERK, and p38 MAPK phosphorylation in response to LPS (Fig. 5), suggesting that MAPKs are involved in the suppression of LPS-induced NF-κB activation by AEGT. Collectively, the proposed action model is presented in Fig. 8.

In addition to inhibition of LPS or CCl_4_-stimulated proinflammatory cytokines by pre-treatment or post-treatment of AEGT *in vitro*, *ex vivo* and *in vivo* (Fig. 3, Fig. 6, Fig. S4 and Fig. S5A–C), AEGT also significantly reversed elevation of proinflammatory cytokines in response to other stimuli, such as TNF-α or IFN-γ (Fig. S3). These findings demonstrated that AEGT exerted prophylactic and curative effects of anti-inflammatory activity and could be widely used as nutritional supplement against inflammatory diseases.

In the human body, liver plays an important role in metabolized the toxic chemicals to nontoxic substances. The harmful toxic chemicals will directly injure the liver by arise the oxidative stress and severe inflammatory response [32]. CCl_4_-intoxicated acute liver injury is a classical system for screening hepatoprotective or agents against oxidative stress or inflammation in experimental animal model. CCl_4_ is catalyzed by a microsomal cytochrome P450-dependent monooxygenase system forming highly reactive trichloromethyl and trichloromethyl peroxy radicals in liver and other organs [33]. These free radicals cause overproduction of reactive oxygen species (ROS) and auto-oxidation of fatty acids, ultimately leading to hepatic damage [33], [34]. Several antioxidants, such as silymarin, Vitamin E and melatonin, ameliorate the impairment of hepatic function [35], [36]. Currently, our previous study demonstrated that AEGT has great ability in protection of H_2_O_2_-induced oxidative DNA-damage and promotion of cell survival at the high concentration (4 mg/ml) of AEGT treatment due to containing polyphenol, flavonid, and ascorbic acid [18]. In the present study, both pre-treatment and post-treatment of AEGT effectively ameliorated the CCl_4_-intoxicated liver architectural and functional damage, such as reversion of CCl_4_-elevated hepatic ALT and AST enzymes (Fig. 7 and Fig. S5D–5I). Further studies are needed to investigate the relevant pathways of antioxidant properties of AEGT involved in hepatoprotective effect against toxic chemicals.

Nowadays, silymarin complex, natural flavonolignans from Silybum marianum seed extract, is known to have anti-inflammatory and hepatoprotective effects, and it is commercially available worldwide in the healthy food market as nutritional supplement for management of acute and chronic liver diseases by inhibiting NF-κB and MAPK/ERK1/ERK2 signaling pathway [37], [38]. However, the poor water solubility of silymarin may lead to poor bioavailability [39]. In contrast, the water-soluble AEGT extracted from aqueous fraction of edible *Gracilaria tenuistipitata* may facilitate bioavailability of AEGT. Furthermore, the edible *G. tenuistipitata* is a mariculture algae in Taiwan from 1961 [40], which is able to be cultivated in a three-dimensional space for large-scale production, therefore, the yield of the *G. tenuistipitata* may be higher than that of the terrestrial plant, such as *Silybum marianum*. Thus, the *G. tenuistipitata* may become an inexpensive resource for commercial purpose of AEGT.

## Conclusion

*In vitro*, *ex vivo* and *in vivo* assays demonstrated that AEGT, an aqueous extract from the edible marine algae *G. tenuistipitata*, significantly reduced the levels of proinflammatory mediators (iNOS and COX-2) and proinflammatory cytokines (TNF-α, IL-1β and IL-6) caused by LPS or CCl_4_. NF-κB and MAPK inactivation by AEGT contributes to these inhibitory effects. It is noteworthy that both preventive and curative effects of the anti-hepatotoxic activity of AEGT *in vivo* was observed in a rat model of CCl_4_-induced liver injury, indicating that AEGT has potential as a nutritional supplement for preventing or curing acute liver damage. Accordingly, future studies are needed to fractionate active ingredients against inflammation.

## Supporting Information

Figure S1 **Characterization of AEGT extract.** The NMR spectrum was identified using Varian Unity-plus 400 MHz FT-NMR. The signals in region I (δH 3.0–4.0) represented the polysaccharide complex. The signals in region II (δH 7.0–7.5) represented the signal of phenoids molecules.
(TIF)

Figure S2 **Effect of AEGT on the cell viability of RAW 264.7 cells.** The RAW 264.7 cells were seeded in a 6-well plate, treated with 400 or 800 µg/ml AEGT for 1 h, and then incubated with 1 µg/ml LPS for 24 h. Treatment of PBS buffer and 1 µM Taxol served as the negative (mock) and positive control, respectively. Cell viability was measured by the flow cytometry using Annexin V/propidium iodide.
(TIF)

Figure S3 **Effect of AEGT on the production and gene expression of proinflammatory cytokines in IFN-γ or TNF-α-stimulated RAW 264.7 cells.** The RAW264.7 cells were seeded in a 24-well plate, treated with 400 or 800 µg/ml AEGT for 1 h, and then incubated with 2.5 ng/ml TNF-α or 200 UI IFN-γ for 24 h. The RNA levels of proinflammatory cytokines induced by TNF-α (A, B and C) and IFN-γ (D, E and F) were analyzed by qRT-PCR, respectively. The efficiency of inhibition was determined as the percent RNA levels relative to those in the cells treated with IFN-γ or TNF-α alone. Error bars indicate the means ± SD of three independent experiments. *P<0.05; **P<0.01.
(TIF)

Figure S4 **Effect of AEGT on the production and gene expression of proinflammatory cytokines in LPS-pre-stimulated RAW 264.7 cells.** The RAW264.7 cells were seeded in a 24-well plate, treated with 1 µg/ml LPS for 6 h, and then incubated with 400 or 800 µg/ml AEGT for 24 h. The RNA (A, B and C) and secreted protein levels (D, E and F) of proinflammatory cytokines were analyzed by qRT-PCR and ELISA, respectively. The efficiency of inhibition was determined as the percent RNA levels relative to those in the cells treated with LPS alone. Error bars indicate the means ± SD of three independent experiments. *P<0.05; **P<0.01.
(TIF)

Figure S5 **Effect of AEGT on CCl_4_-pre-induced acute liver inflammation in Wistar rat.** The Wistar rats were administered CCl_4_ for 1 day and then given the indicated doses of AEGT for 4 days. The rats were sacrificed to collect blood samples and liver sections for inflammatory parameter analysis of secreted protein levels of proinflammatory cytokines including TNF-α (A), IL1-β (B) and IL-6 (C), and biochemical parameter analysis of AST (D) and ALT (E) and histopathological examination [Healthy control (F), CCl_4_ with saline (G), CCl_4_ with 150 mg/kg/day AEGT (H), and CCl_4_ with 300 mg/kg/day AEGT (I)]. Values are presented as the means of five independent experiments. Error bars indicate the means ± SD of three independent experiments. *P<0.05; **P<0.01.
(TIF)
