# Supplementary figures and images for: Aqueous Extract of Gracilaria tenuistipitata Suppresses LPS-Induced NF-κB and MAPK Activation in RAW 264.7 and Rat Peritoneal Macrophages and Exerts Hepatoprotective Effects on Carbon Tetrachloride-Treated Rat
Source: PLoS One. 2014 Jan 27;9(1):e86557. doi: 10.1371/journal.pone.0086557 (PMC3903563; doi:10.1371/journal.pone.0086557)

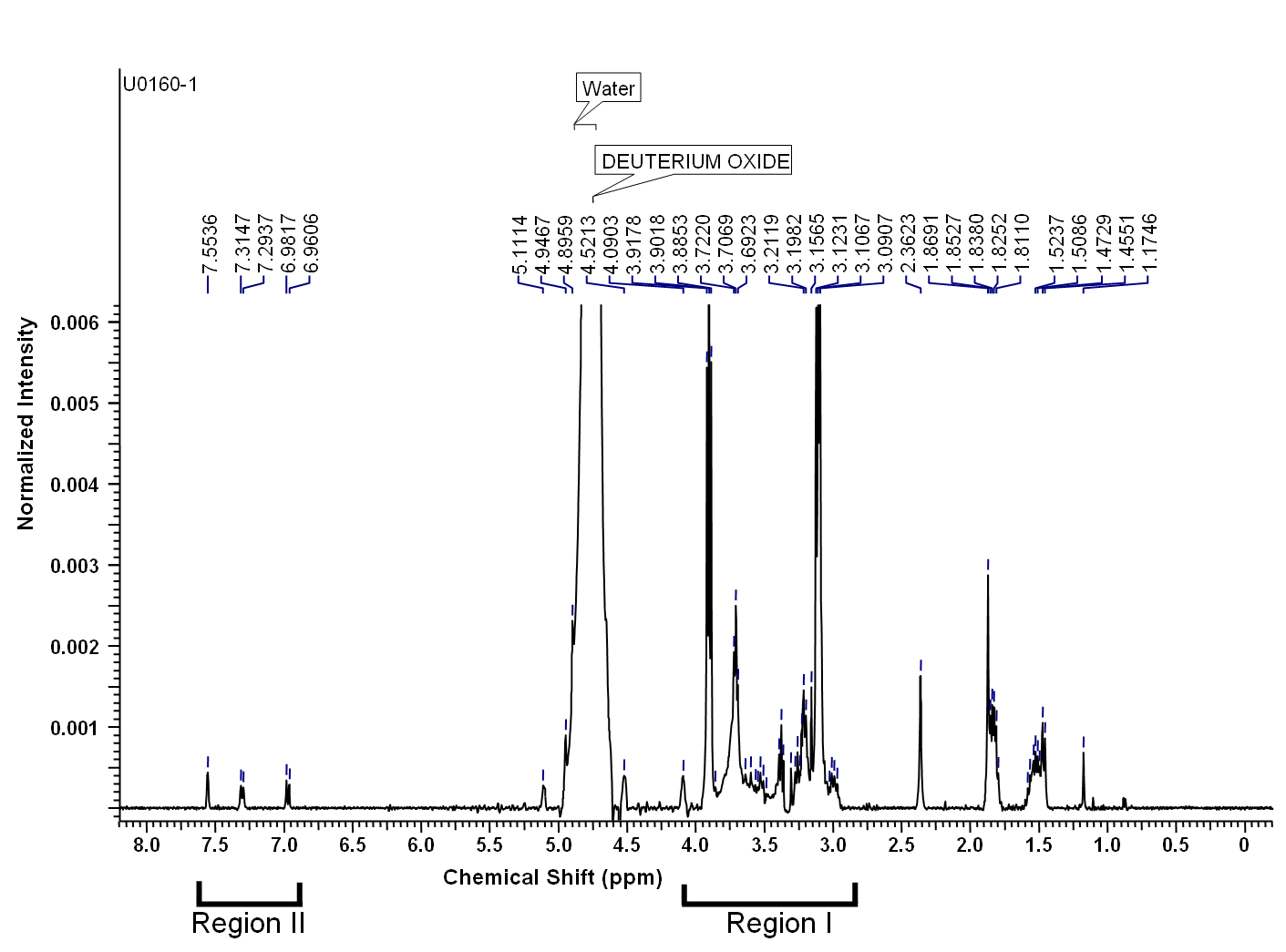

Supplement: Figure S1 — Characterization of AEGT extract. The NMR spectrum was identified using Varian Unity-plus 400 MHz FT-NMR. The signals in region I (δH 3.0–4.0) represented the polysaccharide complex. The signals in region II (δH 7.0–7.5) represented the signal of phenoids molecules. (TIF) [file pone.0086557.s001.tif]

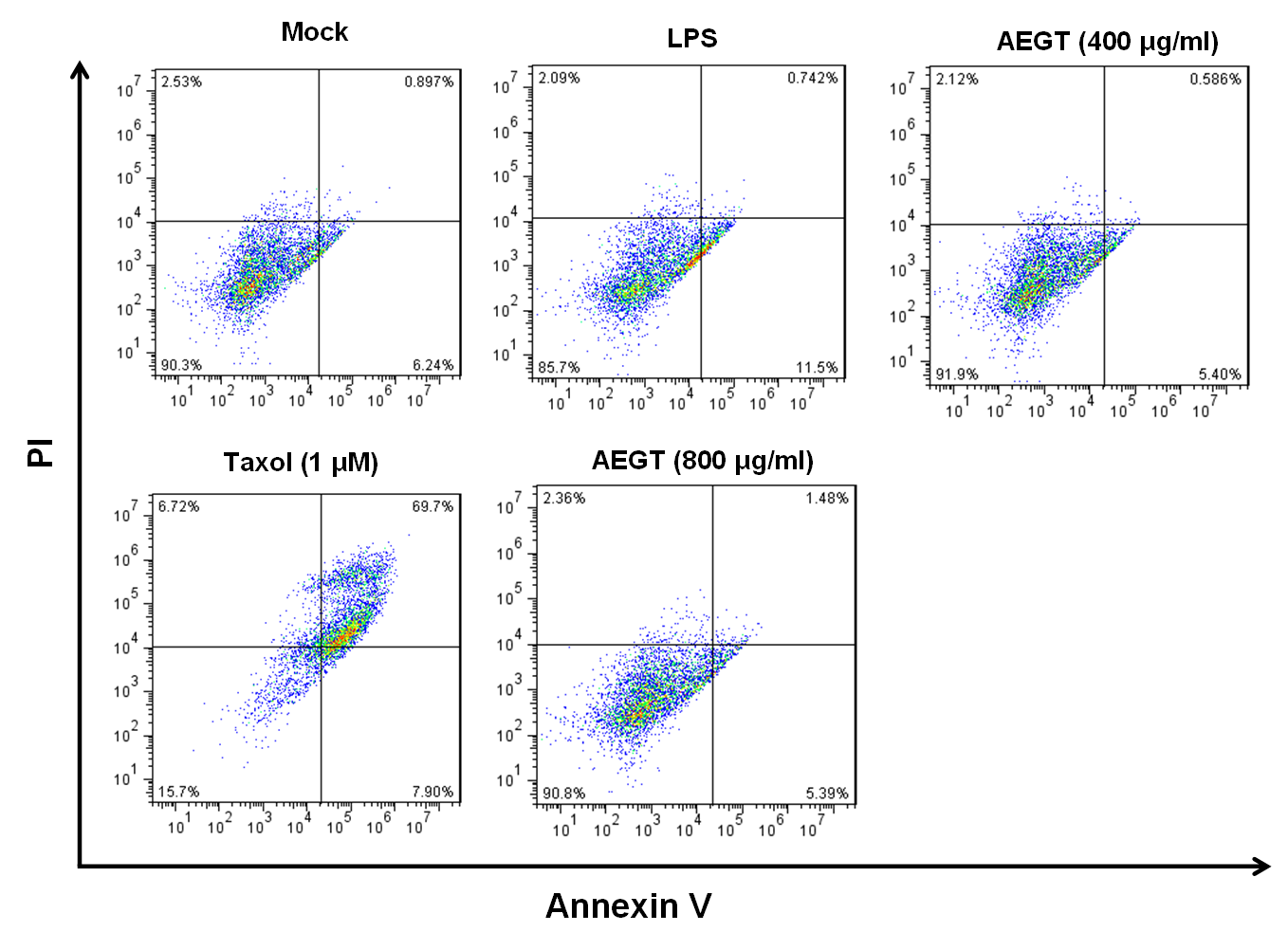

Supplement: Figure S2 — Effect of AEGT on the cell viability of RAW 264.7 cells. The RAW 264.7 cells were seeded in a 6-well plate, treated with 400 or 800 µg/ml AEGT for 1 h, and then incubated with 1 µg/ml LPS for 24 h. Treatment of PBS buffer and 1 µM Taxol served as the negative (mock) and positive control, respectively. Cell viability was measured by the flow cytometry using Annexin V/propidium iodide. (TIF) [file pone.0086557.s002.tif]

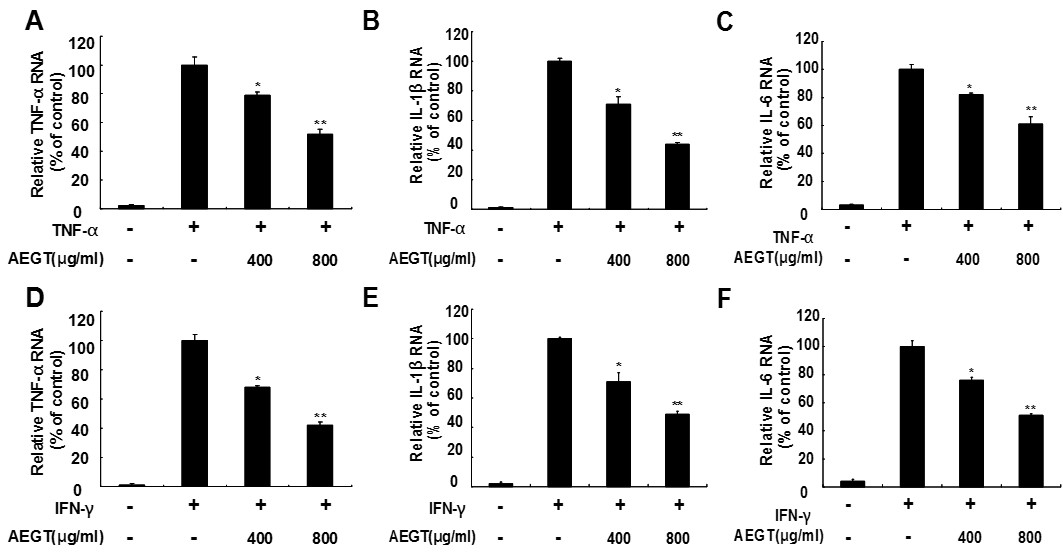

Supplement: Figure S3 — Effect of AEGT on the production and gene expression of proinflammatory cytokines in IFN-γ or TNF-α-stimulated RAW 264.7 cells. The RAW264.7 cells were seeded in a 24-well plate, treated with 400 or 800 µg/ml AEGT for 1 h, and then incubated with 2.5 ng/ml TNF-α or 200 UI IFN-γ for 24 h. The RNA levels of proinflammatory cytokines induced by TNF-α (A, B and C) and IFN-γ (D, E and F) were analyzed by qRT-PCR, respectively. The efficiency of inhibition was determined as the percent RNA levels relative to those in the cells treated with IFN-γ or TNF-α alone. Error bars indicate the means ± SD of three independent experiments. *P<0.05; **P<0.01. (TIF) [file pone.0086557.s003.tif]

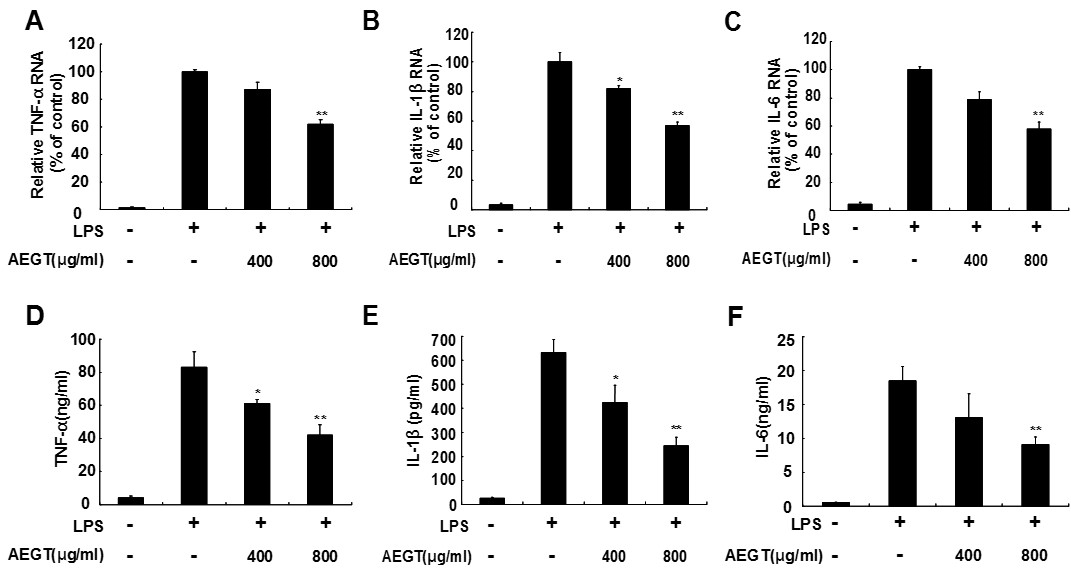

Supplement: Figure S4 — Effect of AEGT on the production and gene expression of proinflammatory cytokines in LPS-pre-stimulated RAW 264.7 cells. The RAW264.7 cells were seeded in a 24-well plate, treated with 1 µg/ml LPS for 6 h, and then incubated with 400 or 800 µg/ml AEGT for 24 h. The RNA (A, B and C) and secreted protein levels (D, E and F) of proinflammatory cytokines were analyzed by qRT-PCR and ELISA, respectively. The efficiency of inhibition was determined as the percent RNA levels relative to those in the cells treated with LPS alone. Error bars indicate the means ± SD of three independent experiments. *P<0.05; **P<0.01. (TIF) [file pone.0086557.s004.tif]

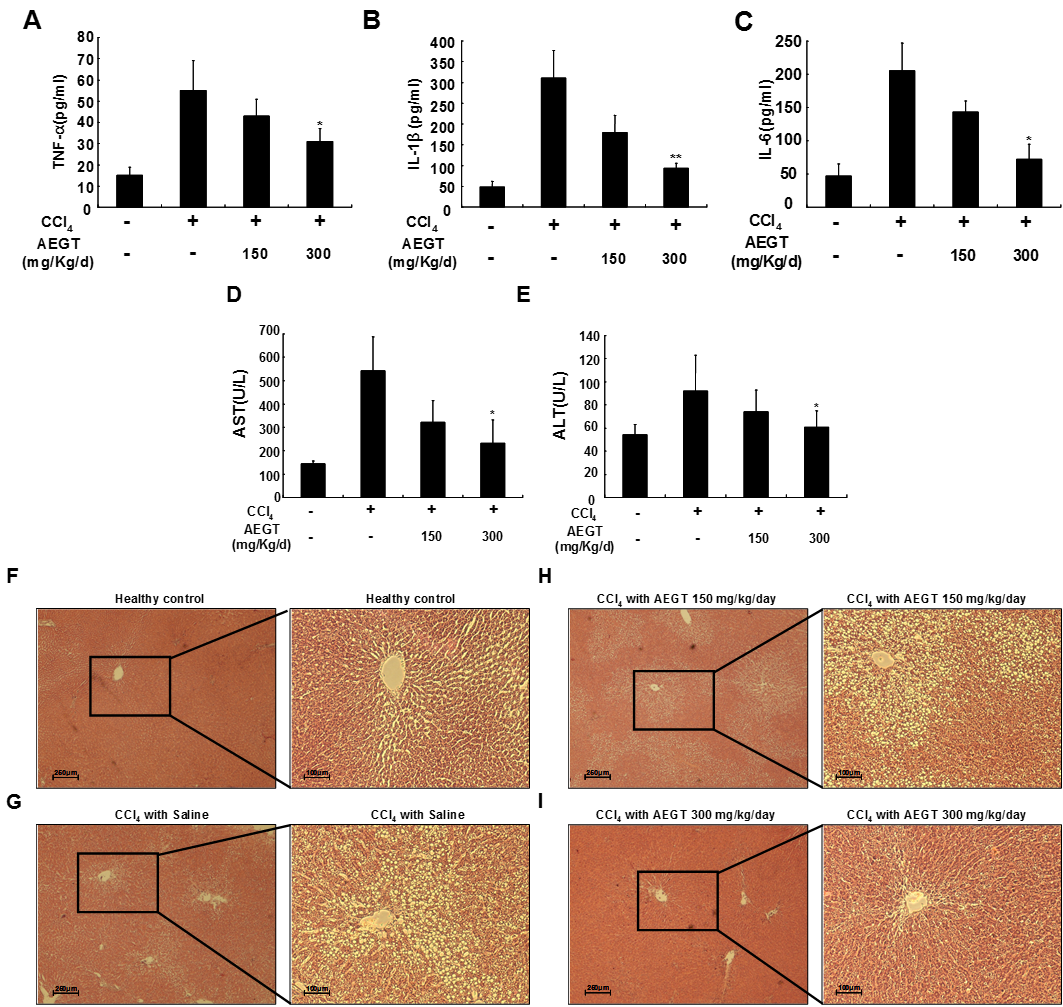

Supplement: Figure S5 — Effect of AEGT on CCl4-pre-induced acute liver inflammation in Wistar rat. The Wistar rats were administered CCl4 for 1 day and then given the indicated doses of AEGT for 4 days. The rats were sacrificed to collect blood samples and liver sections for inflammatory parameter analysis of secreted protein levels of proinflammatory cytokines including TNF-α (A), IL1-β (B) and IL-6 (C), and biochemical parameter analysis of AST (D) and ALT (E) and histopathological examination [Healthy control (F), CCl4 with saline (G), CCl4 with 150 mg/kg/day AEGT (H), and CCl4 with 300 mg/kg/day AEGT (I)]. Values are presented as the means of five independent experiments. Error bars indicate the means ± SD of three independent experiments. *P<0.05; **P<0.01. (TIF) [file pone.0086557.s005.tif]
